# 963. Antibiotic Talk on TikTok: An Opportunity for Patient Education?

**DOI:** 10.1093/ofid/ofab466.1158

**Published:** 2021-12-04

**Authors:** Lauren R Biehle, Emma Evans, Aislinn O’Kane

**Affiliations:** 1 University of Wyoming, Laramie, Wyoming; 2 Department of Veterans Affairs, Corpus Christi, Texas

## Abstract

**Background:**

Antimicrobial resistance is increasing at an alarming rate. Patient education is a critical component of stewardship and many patients access resources online. TikTok is a video-sharing social media platform with 700 million monthly users and contains videos that discuss health information. The objective of this study was to evaluate antibiotic-themed TikTok videos for their validity and reliability.

**Methods:**

In March 2021, a search on TikTok using the term “antibiotics” was performed and the top 300 consecutive videos were identified. Data collected included: number of likes, associated disease state, medications, educational aim, mention of COVID-19, and if performed by a healthcare professional (HCP). Non-English videos were excluded. The DISCERN score was used to evaluate all videos for reliability.

**Results:**

The first 300 consecutive videos were assessed using the DISCERN score. Of the 300 videos, most (n=224) were not created by HCPs (non-HCPs). The number of “likes” per video ranged from 1 like to 2 million likes with a mean of 34,949 ± 143,482. Videos produced by HCPs were significantly more valid and reliable (mean DISCERN score of 1.65 vs 1.17, p < .00001) than non-HCPs. They were found to be more relevant (p< .00001), have clearer aims (p< .00001), and were more balanced/unbiased (p=.00188). Videos created by HCPs were more likely to have an educational focus (p< .0001). There was no difference between groups in clarity of sources utilized or risk/benefits discussed of each treatment. Across all videos, the most common disease states mentioned were urinary tract infection, skin and soft tissue infection, and upper respiratory tract infection. Natural products, penicillins, and sulfa antibiotics were the most commonly discussed medications.

**Conclusion:**

Videos created by HCPs were significantly more valid and reliable than those created by non-HCPs. The videos created by HCPs were also more likely to have clear aims and be more relevant. However, the majority of the videos evaluated were created by non-HCPs. It may be beneficial for HCPs to provide TikTok videos that are valid and reliable for patient education.

**Disclosures:**

**All Authors**: No reported disclosures

